# Supplementary material for: Vitamin D Modified DSS-Induced Colitis in Mice via STING Signaling Pathway
Source: Biology (Basel). 2025 Jun 18;14(6):715. doi: 10.3390/biology14060715 (PMC12190092; doi:10.3390/biology14060715)
Supplement: Supplementary file 1 [file biology-14-00715-s001.zip › Supplementary Materials S1: 16s-report of gut microbiota/Groups/Lefse_Analysis/biomarkers_raw_images/1_d__Bacteria-p__Desulfobacterota-c__Desulfovibrionia.pdf]

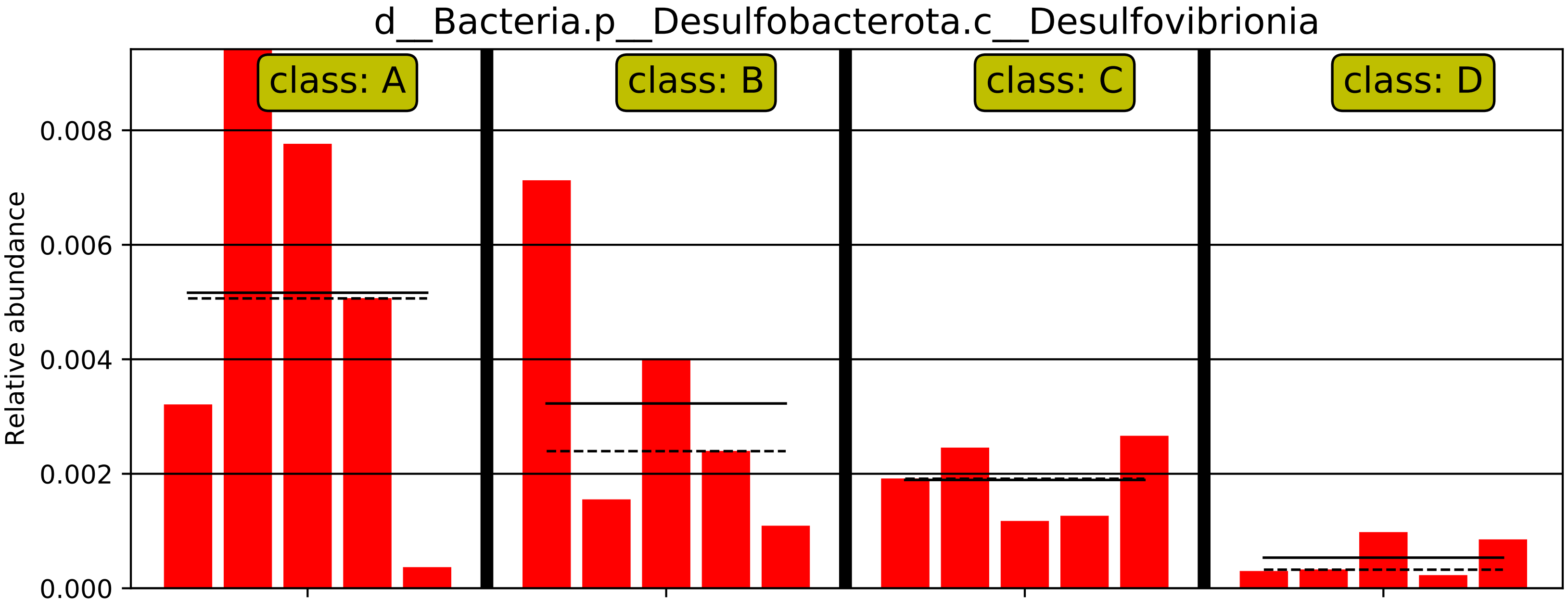

d__Bacteria.p__Desulfobacterota.c__Desulfovibrionia
class: A
class: B
class: C
class: D
Relative abundance
0.008
0.006
0.004
0.002
0.000
